# Using geomorphological variables to predict the spatial distribution of plant species in agricultural drainage networks

**DOI:** 10.1371/journal.pone.0191397

**Published:** 2018-01-23

**Authors:** Gabrielle Rudi, Jean-Stéphane Bailly, Fabrice Vinatier

**Affiliations:** 1 LISAH, Univ Montpellier, INRA, IRD, Montpellier SupAgro, Montpellier, France; 2 G-Eau, Univ Montpellier, AgroParisTech, CIRAD, IRD, IRSTEA, Montpellier SupAgro, Montpellier, France; 3 AgroParisTech, Paris, France; Universidade Federal de Goias, BRAZIL

## Abstract

To optimize ecosystem services provided by agricultural drainage networks (ditches) in headwater catchments, we need to manage the spatial distribution of plant species living in these networks. Geomorphological variables have been shown to be important predictors of plant distribution in other ecosystems because they control the water regime, the sediment deposition rates and the sun exposure in the ditches. Whether such variables may be used to predict plant distribution in agricultural drainage networks is unknown. We collected presence and absence data for 10 herbaceous plant species in a subset of a network of drainage ditches (35 km long) within a Mediterranean agricultural catchment. We simulated their spatial distribution with GLM and Maxent model using geomorphological variables and distance to natural lands and roads. Models were validated using k-fold cross-validation. We then compared the mean Area Under the Curve (AUC) values obtained for each model and other metrics issued from the confusion matrices between observed and predicted variables. Based on the results of all metrics, the models were efficient at predicting the distribution of seven species out of ten, confirming the relevance of geomorphological variables and distance to natural lands and roads to explain the occurrence of plant species in this Mediterranean catchment. In particular, the importance of the landscape geomorphological variables, ie the importance of the geomorphological features encompassing a broad environment around the ditch, has been highlighted. This suggests that agro-ecological measures for managing ecosystem services provided by ditch plants should focus on the control of the hydrological and sedimentological connectivity at the catchment scale. For example, the density of the ditch network could be modified or the spatial distribution of vegetative filter strips used for sediment trapping could be optimized. In addition, the vegetative filter strips could constitute new seed bank sources for species that are affected by the distance to natural lands and roads.

## Introduction

Agricultural drainage networks of headwater catchments are of high ecological value. They represent interfaces between anthropized and natural landscapes because they are mainly composed of linear hydro-agroecological infrastructures of anthropogenic origin [1] that are located at the boundary of fields and that are poorly constrained by crop technical operations. Their network design is generally in accordance with natural landforms and thalwegs [2]. They are also intermediary ecosystems between terrestrial and aquatic landscapes because they experience intermittent flooding and temporary waters [3] and are prone to upstream-downstream gradients. These drainage networks host various plant communities [3–5] that interact with biotic and abiotic components of the entire ecosystem [6,7]. Ditch vegetation may enhance ecosystem services including bank erosion mitigation [8], pesticide or nutrient retention [9–13], and groundwater recharge. On the other hand, the ditch vegetation may also provide disservices including the impairment of network water transport [14] and the enhancement of weed dispersal [15]. Estimating the services and disservices provided by drainage networks clearly requires the characterization of the spatio-temporal distribution of plant species along the networks.

Niche theory relies on the premise that plant species establish in specific environmental conditions [16,17]. These environmental conditions are sometimes called the fundamental niche [18]. Even when the niche is favourable for a species, other exogenous drivers such as niche accessibility [19] and human practices (especially in cultivated lands) [20–22] may impact the distribution of species.

In rain-fed agricultural headwater catchments under semi-arid climate, the factors that affect the occurrence of plant species in ditches indirectly depend on the local and landscape geomorphology. In this paper, we consider that the local geomorphology encompasses the close environment of the ditch, while the landscape geomorphology encompasses a broader environment surrounding the ditch. The local geomorphology, such as the slope in the ditches, and the landscape geomorphology, that determines the surface area drained by a section of ditch, impose varying hydraulic and hydrological conditions [23]. These variables determine the strength and duration of the exposure of plants to the water dynamics in surface and in the soil [24], which is crucial to explain the spatial distribution of species in water-limited ecosystems [25]. The strength of the association between geomorphology and plant spatial distribution is plant-specific, i.e., it depends on the facultative/obligate wetland nature of the considered species [26]. All hydrological and hydraulic processes in such systems are structured along an upstream-downstream gradient due to the growing surface area drained by the ditches from upstream to downstream and the dendritic structure of the drainage network. Consequently, the relative distance to the outlet may be an indirect indicator for describing the hydraulic conditions in a network. Moreover, in rivers and streams, geomorphology partly controls where sedimentation and erosion occur [27], and affects superficial bed soils by depositing or removing sediment layers. The same mechanisms are expected to take place in drainage systems in headwater catchments and the spatial distribution of valleys and ridges at different spatial scales in the catchment might determine the importance of deposited materials in the network. Lastly, the global sun exposure of the hillslopes and the solar radiation are known to affect the temperature conditions in streams and the plant responses to light [28]. Geomorphological variables were found to be good predictors of aquatic plant assemblages by Manolaki and Papastergiadou [29], who focused on the distribution of macrophytes in Mediterranean rivers. Maheu-Giroux and de Blois [30] emphasized that although the geomorphology of ditches may be a major factor explaining the spatial distribution of species, geomorphology is seldom considered in studies concerning linear hydro-agroecological infrastructures.

Niche accessibility is defined by the composition and spatial patterns of landscape features, and these composition and structure of landscapes may influence species richness [31,32]. In agricultural landscapes, the surrounding natural lands may represent significant propagule sources [33]. Van Dijk et al. [34] showed that the distance to natural reserves can influence community richness. In addition, roads may help transport plant propagules that may colonize roadsides and field boundaries [35] such as ditches. As is the case in rivers, niche accessibility in agricultural drainage networks is related to the surface area drained by the ditch and the length of the upstream network, because some plant propagules, whether hydrochorous or not, are transported along an upstream-downstream gradient [36]. When possible, niche accessibility should be analysed jointly with the dispersal abilities of the species because both define the connectivity between habitats for a given species. In agricultural landscapes, niche accessibility is therefore a major factor explaining plant patterns.

Compared to agricultural fields, ditches in agricultural headwater catchments are generally subjected to infrequent but regular management practices [14] but the practices may differ over time. In the long term, the effects of different practices may interact, because the effects of an agricultural practice can persist for several years [37]. The effects of some agricultural practices on plant distribution and richness have been investigated for field boundaries [4,38,39] but led to various conclusions. Additionally, little is known about how drainage networks might be managed to optimize the trade-offs between ecosystem services and disservices in Mediterranean agricultural areas.

Disentangling the influences of these different exogenous factors on plant distribution could facilitate agro-ecological engineering. The agro-ecological measures for this type of ecosystem might be of several types. Some authors [14,38] have shown that some local management practices, such as mowing for example, could modify the composition and distribution of the plant communities in the ditches in the medium term. Dollinger et al. [7] explained that the modification of the ditch morphological characteristics can have an impact on the abiotic properties of the ditches, and this could also have an impact on ditch communities. Cordeau et al. [40] determined that the establishment of sown grass strips had an impact on the spatial distribution of vegetation in field boundaries. Lastly, some other agro-ecological measures can be used to optimize ditch networks [41,42] such as restoring, creating or suppressing some ditches. This would result in changing the density of the network and modifying the surface area drained per section of ditch and the peak discharges [42]. Before applying any type of measure to control the occurrence of plant species, it is essential to know more about the factors that affect their spatial distribution in a whole network of ditches in a Mediterranean landscape. Indeed, it has been proven that years of agri-environmental measures (essentially local management measures) in the Netherlands was not totally successful in restoring the biodiversity on ditch banks [43]. This has led some authors to emphasize the role of other factors that have not been considered at first in the spatial distribution and composition of plant communities, such as connectivity or regional factors [33,43,44]. This example illustrates the importance of understanding how exogenous factors affect the spatial distribution of plant species in ditches under a Mediterranean semi-arid climate.

For the study of exogenous factors that structure plant community composition, models that predict the presence or presence/absence of species (spatial distribution models or SDMs) are often useful [45]. SDMs have often been used to identify areas where specific plants and invasive species have the capacity to develop [46]. SDM are largely used in the literature on species distribution ecology [47] because of their increased predictive capacities, the possibility of cross-validation, and finally the use of non-categorical variables, in comparison to other recent multivariate approaches such as Permanova [48] or structural equation modelling [49].

This paper presents a catchment-scale analysis of plant patterns along an agricultural drainage network composed of ditches. Our hypothesis is that variables based on geomorphology and distance to natural lands and roads have the ability to predict the occurrence of plant species in a network of ditches in a semi-arid climate. Specifically, geomorphology could have an important effect due to the relationship between this geomorphology and the water regime under this type of climate. We also attempt to understand the origin of the part of the variability that cannot be explained by geomorphology and distance to natural lands and roads.

## Materials and methods

### Study area

The study area is a 6.4-km^2^ headwater catchment named « Bourdic » in southern France, located northeast of Béziers city. The main towns of the area are Alignan-du-Vent and Roujan. Approximately 74% of the catchment is agricultural (mainly vineyards), and 26% is semi-natural (mainly woodlands and shrubs). The mean annual temperature is 14°C [23]. The catchment has a Mediterranean climate with heavy rainfalls causing significant Hortonian runoff [50]. The precipitation generally ranges from 600 to 800 mm per year [2], but some years can be drier. The rainfall regime is intermittent, with a dry period from April to October and heavy rainfalls in Autumn and Spring. Annual potential evapotranspiration is about 1100 mm [23]. Formally, the climate in the area is at the boundary between the sub-humid and the semi-arid climate, although the trends in the recent years tended to classify it as a semi-arid climate [51]. This is this last definition that we conserved for the study. The altitude ranges from 55 m a.s.l. at the outlet at the northeast to 128 m a.s.l. at the northwest. The following geomorphological units can be differentiated: an upstream-downstream succession of plateaus, steep slopes, and valley plains. Slopes up to 8% occur in the east, west and centre, and plains occur mainly in the north. The geomorphological units are associated with different soil typologies and sedimentary layers resulting from various erosion/redeposition mechanisms [52].

The man-made drainage network consists exclusively of agricultural and roadside ditches. The agricultural ditches were created over the centuries by farmers for the collection of water during intense rainfalls and for soil conservation. The network is 76 km long, and the density of the network is thus 119 m/ha. A survey in a subcatchment of 1 km^2^ conducted by Levavasseur et al. [42] showed that 75% of the ditches had an upper width between 50 and 120 cm and a depth varying between 30 and 80 cm. The ditches form a directed hierarchical network (a dendritic structure). The drainage network is connected to a single outlet in the east of the study area. Note that in this paper, the word “ditch” refers to a homogeneous portion of network as regards with its properties (width, depth, sediment layer…), that can be variable in length, as defined by Lagacherie et al. [53], while a “ditch section” refers to a subset of the ditch according to the resolution of the study (2 m x 2 m in our case).

The Mediterranean vegetation growing on the bed of ditches (vegetation along the banks was not part of the study) included herbaceous grasses and forbs, and shrubs. The ditches are managed by farmers or local authorities, and vegetation management includes burning, herbicide application, and mowing [14]. The ditches are dredged when the hydraulic capacity requires restoration [14]. The frequency of management is 1.3 times per year in the study area [23] but varies with typology of the practice, period, and location in the network.

The methods followed these three main steps: (i) a survey of the occurrence of ditch plants along the network and the characterization of the ditch network (ii) the assessment of the spatial autocorrelation of plant patterns using Moran indices and spatial sorting bias in order to select non-correlated samples (iii) the use of a generalized linear model (GLM) and of a species distribution model (Maxent) to predict the occurrence of each species.

### Plant species distribution survey

Free access to private properties located in agricultural areas is permitted by French legislation, although landowners can apply for an exception as described in Article 647 of the Civil Code.

The following 10 herbaceous species were surveyed along the network: wild asparagus (*Asparagus acutifolius)*, sand-couch (*Elytrigia juncea)*, common horsetail (*Equisetum arvense)*, purple loosestrife (*Lythrum salicaria)*, water mint (*Mentha aquatica)*, round-leafed mint (*Mentha suaveolens)*, shrubby blackberry (*Rubus fruticosus)*, curly dock (*Rumex crispus)*, round-headed club-rush (*Scirpoides holoschoenus)*, and Johnson grass (*Sorghum halepense)*. These species were selected mostly because they differ in their sensitivity to various water regimes, and consequently were expected to be predicted heterogeneously by the different geomorphological variables. They were also selected because of their relative abundance and relative ease of identification. The studied species were classified according to their niche characteristics (Table 1), using Julve [54].

**Table 1:** Ecological optima, spatial autocorrelation (SAC) critical distances, frequency of occurrence of the 10 species after consideration of SAC and spatial sorting bias (SSB).

Species	Ecological optimum					SAC	Frequency after SAC			SSB after SAC
	Light	Soil moisture	pH	Texture	Organic matter	Moran critical distance (m)	Number of "presence" rasters	Number of "absence" rasters	Frequency (%) of presence rasters	
***Asparagus acutifolius***	5	3	6	5	2	14	159	2 165	6.8	0.8
***Elytrigia juncea***	9	5	7	5	1	22	416	1 101	27.4	0.9
***Equisetum arvense***	7	3	5	3	3	30	279	786	26.2	0.8
***Lythrum salicaria***	7	5	6	1	8	26	133	1 055	11.2	0.8
***Mentha aquatica***	5	6	5	1	8	26	25	1 158	2.1	0.6
***Mentha suaveolens***	8	5	6	2	8	22	135	1 246	9.8	0.8
***Rubus fruticosus***	5	4	2	3	5	18	787	1 266	38.3	0.9
***Rumex crispus***	7	5	5	1	8	10	147	3 026	4.3	0.9
***Scirpoides holoschoenus***	8	6	7	1	9	18	185	1 601	10.4	0.8
***Sorghum halepense***	8	4	7	4	3	14	65	2 237	2.8	0.6

Ecological optima are based on a 1–9 scale from a minimum to a maximum. Texture ranged from clay (1) to rocks (9). SAC distances were based on Moran indices. The frequency of occurrence of species was given after considering SAC.

The surveys were conducted in July-August 2013 according to a non-destructive sampling procedure using GPS with an Android self-developed application [55]; this enabled a location accuracy of 2 m. Agricultural ditches, including roadside ditches, were part of the study. Thirty-five kilometres of the drainage network (46%) were surveyed for presence/absence of the species. The remaining ditches were excluded from the analysis because surveying them was impractical or because recent management practices impaired the identification of the species. After the survey, the georeferenced data were exported in a shapefile data format with line features.

### Ditch network characterization

The Bourdic drainage network, which is mainly composed of ditches, was first digitized in 2008–2009 based on aerial images [23]; the information was regularly updated with field surveys during the 2012–2013 period. During these field surveys, the depths and the upper and lower widths of ditch cross sections were measured at 675 locations. We assumed that the ditch cross-sections did not change significantly between survey times. Each ditch section was then assigned with the presence/absence of plant species and with cross-sections measures. The ditch network was finally rasterized on a grid with a resolution of 2 m; this resolution was selected based on the precision of the GPS used in the field survey and on the resolution of the digital terrain model (DTM).

### Plant species spatial autocorrelation

The spatial pattern of each species was analysed separately in order to determine the range of spatial autocorrelation (SAC); this was done to exclude samples that were spatially autocorrelated from further analyses and to build a proper sampling design for model estimation [56]. If spatially autocorrelated samples are not excluded, the importance of environmental variables can be overestimated [57]. This analysis was conducted with Moran indices [58] based on stream distances. For each of the presence rasters, a matrix was created by counting presence points in the neighbourhood at several ranges of distances (from 2 m to 100 m). The Moran index corresponds to the ratio between covariance for neighbouring points and total variance [59]. For each species, Moran indices were then converted to Z-scores, and the critical distance indicating a significant spatial autocorrelation was determined. Finally, we resampled the presence rasters in order to space them with the critical distance established from Moran indices and to exclude spatial autocorrelation effects from further analysis. Critical distances calculated from Moran indices and the number of presence and absence pixels for each plant species after considering SAC were reported in Table 1. The consideration of the critical distance calculated with Moran indices drastically reduced the number of presence and absence rasters for each species.

### Geomorphological variables and distance to natural lands and roads

The landscape geomorphological variables included the distance to the outlet (**Doutlet**), the drained surface area (**Drain**), the Multiresolution Index of Valley Bottom Flatness (**Mrvbf**), and the sun exposure of the slopes (**Northness**). The local geomorphological variables were the slope (**Slope**) and solar radiation (**Solar**). The difference between local and landscape geomorphological variables is that the local variables encompass the close environment of a ditch, while the landscape variables encompass a broader environment surrounding the ditch. Distance to natural lands (**Dnat**) and distance to roads (**Droad**) were also chosen as explanatory variables (Table 2).

**Table 2:** Summary of acronyms used for explanatory variables and type of variables.

Variable	Acronym	Type of variable
Distance to Outlet	Doutlet	Geomorphological (Landscape)
Drained Surface Area	Drain	Geomorphological (Landscape)
Multi-resolution Valley Bottom Flatness	Mrvbf	Geomorphological (Landscape)
Northness	Northness	Geomorphological (Landscape)
Slope	Slope	Geomorphological (Local)
Solar Radiation	Solar	Geomorphological (Local)
Distance to Natural Areas	Dnat	Distance to Land-use
Distance to Roads	Droad	Distance to Land-use

**Fig 1:**
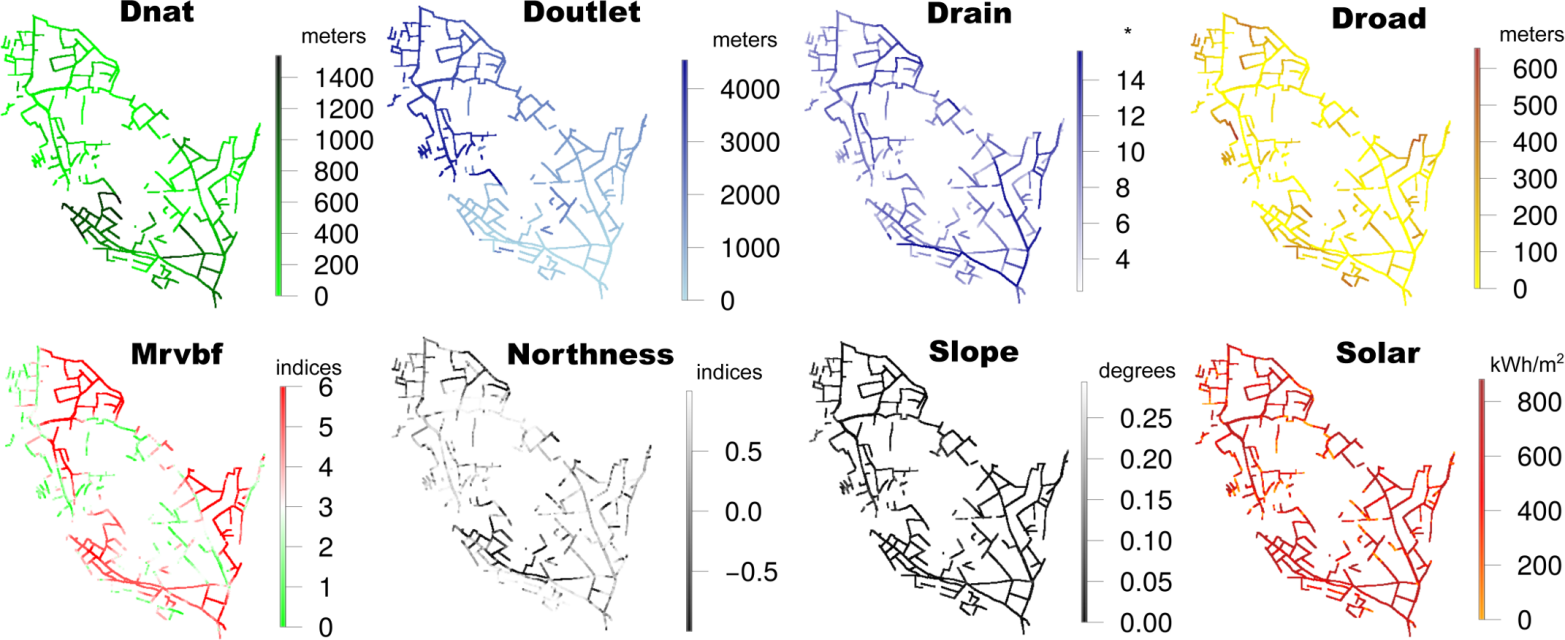
Spatial variability for each explanatory variable at the catchment scale. **Dnat** is the distance to natural areas. **Doutlet** is the distance to the outlet. **Drain** is the Drained Surface Area. **Droad** is the distance to roads. **Mrvbf** is the Multi-resolution Valley Bottom Flatness. **Northness** is the exposure of slopes in relation to an East-West axis. **Slope** is the local slope of a ditch section. **Solar** is the direct potential incoming Solar Radiation. * Drain is expressed in log (meters^2^ +1).

The six geomorphological variables were all derived from 2-m-resolution Digital Terrain Model (DTM) grids and Digital Surface Model (DSM) grids obtained from LIDAR surveys conducted in 2001 [60]. We considered that the geomorphology did not change significantly between 2001 and 2013. Indeed, the study area is mainly covered with vineyards and no major reorganization of the landscape took place between 2001 and 2013. The major driver of landscape reorganization in the region during this period was urbanization, and it was not significant in the study area. Regarding the morphology of the ditch network as such, the geometrical properties were updated in 2012–2013 with field surveys, as was described above. A “ditch section” consisted of a 2m*2m section area (size of the pixel). A DTM pre-process consisted of stream-burning [61] the rasterized drainage network in the DTM and DSM in order to correctly force preferential water paths through the network of ditches. We hypothesized that the distance to outlet (**Doutlet**), the drained surface area (**Drain**) and the local slope of the ditch (**Slope**) accounted for the hydraulic and hydrological regimes, and also indirectly for soil hygromorphy, in a section of ditch. Indeed, the water regime is the major factor explaining plant spatial patterns in riparian areas [62,63] and then possibly explain the spatial distribution of plants in Mediterranean ditches. **Doutlet** was then calculated with 2-m accuracy, because it was computed as the shortest path via the gridded network from a ditch section to the outlet. **Drain** was the whole upstream area drained by a ditch section as computed with the usual D8 algorithm [64] on the stream-burned DTM. **Slope** represented the rasterized difference in elevation between ditch terminal nodes divided by ditch polyline lengths. **Mrvbf** is an indicator for soil composition and soil hygromorphy. More specifically, it is a topographic index that enables the mapping of valley bottoms at a range of scales [65]. **Mrvbf** is usually used to locate areas of water basins and deposited materials. **Mrvbf** index values < 0.5 indicate non-valley areas, values between 0.5 and 1.5 indicate steep valley bottoms, and values > 1.5 indicate large valley areas [65]. **Northness** and **Solar** accounted for sun exposure at different spatial scales. **Northness** was the global sun exposure in relation to an East-West reference axis. Solar radiation (**Solar**) was the direct potential incoming solar radiation. A solar constant of 1360.7 kWh.m^-2^ was chosen based on the characteristics of the study area, and 70.1% was selected for the lumped atmospheric transmittance. The dust factor was 100 ppm. Direct Solar radiation was calculated over a 4-month period from March to June. Because **Solar** was calculated from the DSM, shade effects from vegetation were considered jointly with relief effects. The shade effect could be calculated for adjacent pixels but could not be easily calculated for the pixel producing the shade. Consequently, we attributed an incoming solar radiation value of 0 to pixels covered by tall vegetation, i.e., for pixels with an elevation difference of at least 1.5 m between DSM and DTM. We also hypothesized that distance to natural areas would be important because they are potential sources of seeds, therefore we included distance to natural areas (**Dnat**) in our analysis. Furthermore, distance to roads (**Droad**) was also included because roads are important seed dispersal vectors in agricultural landscapes and we hypothesized that they would be important for structuring plant communities in agricultural ditch networks. These variables were created from a land-use map established from a satellite image covering the study area in 2014. They were calculated as the shortest path between pixel centres located on a ditch and those located on natural lands or roads. Because some ditches were located on catchment boundaries, we extended the land use map footprint to 200 m in order to avoid edge effects. The spatial patterns and variability of the height variables are presented in Fig 1.

We tested the correlations between variables because multicollinearity implies that partial regression coefficients might be imperfectly assessed [66]. Because all variables could be considered continuous, a Pearson product-moment correlation test was used. No strong collinearity among variables was detected because all correlation coefficients were below to 0.6 [39,66].

### Data analysis

#### Model selection procedure and importance of variables

The occurrence of the 10 plant species in the ditch network was modelled using the 6 geomorphological parameters and the distance to natural areas and distance to roads as explanatory variables. We only selected prospected parts of the network where presence/absence had been determined. Because different models may differ in their performance depending on the application [67,68], we used two models: (i) a regression model, i.e., the GLM assuming a binomial distribution of values using a logit link [69]; (ii) a SDM, the non-linear maximum entropy model (Maxent) [70]. The GLM uses presence/absence data, but Maxent model uses presence-only data. We selected these models because they have been reported to be among the best distribution models [67,71]. The explanatory variables were rescaled between [0–1] to make their weights comparable during the modelling procedure.

For each plant species and each model, we performed a k-fold cross-validation (k = 4). The number of presence data for each plant species in each learning and validation sample was greater than 10 pixels. Area under the curve (AUC) [72], a threshold-independent statistic, was used to assess the fitting of the different models for each validation sample and each species. A mean AUC value was calculated for the four validation samples. An AUC value ≤ 0.5 indicates that the model does not perform better than a random model; values between 0.5 and 0.7 indicate a low predictive ability; values between 0.7 and 0.9 indicate a good predictive ability; and values > 0.9 indicate an excellent predictive ability [72]. The use of AUC in evaluating the performance of species distribution models has been criticized [73] and particularly because the AUC values are sensitive to the spatial extent used to select background points. To that end, we also calculated the "spatial sorting bias" (ssb), i.e. the difference between the distance from testing-presence to training-presence and the distance from testing-absence to training-presence points, for each species, following Hijmans [74]. A ssb close to 0 means a strong bias whereas a ssb close to 1 means an absence of bias. Before calculating the ssb, the data were spatially resampled to remove SAC by using Moran's autocorrelation indices calculated along the linear networks. Then ssb was calculated for each species after resampling. We could not apply the pairwise distance sampling algorithm proposed by Hijmans [74] due to the specific structure of our dataset (network structure). We also added the accuracy measures derived from the confusion matrices, such as the Positive Predictive Value (PPV), the Negative Predictive Value (NPV) and the Overall accuracy, as proposed by Liu et al. [75]. The predicted values were thresholded using the maximum Kappa value [76] to calculate the confusion matrices. For each explanatory variable, we then determined its relative importance for Maxent, and the value of the coefficients for GLM (positive or negative) for each species. For GLM, because explanatory variables were all converted to a [0–1] scale, absolute values of regression coefficients also represented their relative importance. For GLM, only coefficients with p-values < 0.05 were displayed.

#### Spatial patterns of false positive and false negative predictions

The modelled distributions of species in the validation sample were calculated as continuous probability values. These probabilities were transformed into binary scores using a threshold value calculated from maximum Kappa values [76]. For Maxent, one map for false positive predictions and another map for false negative predictions, with compiled results for the 10 species, were created to identify areas with low prediction scores common to several species.

#### Tools used for analysis

Maps displaying spatial patterns of explanatory variables and false positive/false negative predictions were created using QGIS [77]. All analyses were conducted with R [78]. All explanatory variables were calculated using the RSAGA package [79]. The pre-modelling preparation of data relied on the R Spatstat package [80], R Raster Package [81], R Maptools Package [82], R Rgdal package [83], R sp package [84], R Rgeos package [85], R pbapply package [86], and R igraph package [87]. The R Dismo package [88] was used for the modelling step. Note that the Maxent model was run with the default features, i.e. the convergence threshold at 0.00001 and the maximum number of iterations at 5000.

## Results

SAC varied among species and ranged from 10 to 30 m (Table 1). The critical distances were largest for *E*. *arvense*, *L*. *salicaria*, and *M*. *aquatica*, and were smallest for *R*. *crispus*, *A*. *acutifolius*, and *S*. *halepense*. All ssb calculated after considering SAC (Table 1) were equal to or above 0.6, meaning that the spatial sorting bias was low in our case-study.

The mean AUC values were generally greater for Maxent models than for GLMs (Table 3). The order of species according to AUC values was roughly similar for the two models. Maxent mean AUC values ranged from 0.92 for *M*. *aquatica* to 0.67 for *R*. *fruticosus*, and the standard deviations were moderate (≤0.05) for all species models except for *S*. *halepense* (Sd = 0.11). Maxent species models with the highest mean AUC values (≥ 0.90) were those for *M*. *aquatica* and *L*. *salicaria*. The only Maxent species model with a low mean AUC value (< 0.70) was that for *R*. *fruticosus*. GLM mean AUC values ranged from 0.86 for *L*. *salicaria* to 0.61 for *R*. *fruticosus*, and the standard deviations were moderate (≤0.05) for all species except for *M*. *aquatica* (Sd = 0.09). GLM species models with the highest mean AUC values (≥ 0.85) were those for *L*. *salicaria* and *A*. *acutifolius*. GLM species models with the lowest mean AUC values (< 0.70) were those for *R*. *fruticosus*, *S*. *halepense*, *E*. *juncea*, and *M*. *suaveolens*.

**Table 3:** Mean area under the curve (AUC) values and three metrics derived from confusion matrices with GLM and Maxent model for each species. Standard deviation issued from the cross-validation procedure were represented by values in brackets.

Species	AUC		Positive Predictive Value		Negative Predictive Value		Overall accuracy	
	Maxent	GLM	Maxent	GLM	Maxent	GLM	Maxent	GLM
***Asparagus acutifolius***	0.85 (0.03)	0.85 (0.04)	0.84 (0.04)	0.87 (0.05)	0.78 (0.07)	0.75 (0.05)	0.78 (0.06)	0.76 (0.05)
***Elytrigia juncea***	0.72 (0.03)	0.62 (0.02)	0.76 (0.08)	0.74 (0.04)	0.54 (0.05)	0.48 (0.06)	0.60 (0.02)	0.55 (0.03)
***Equisetum arvense***	0.89 (0.01)	0.79 (0.02)	0.84 (0.09)	0.75 (0.10)	0.86 (0.04)	0.79 (0.07)	0.85 (0.02)	0.78 (0.03)
***Lythrum salicaria***	0.90 (0.02)	0.86 (0.01)	0.80 (0.02)	0.90 (0.03)	0.85 (0.04)	0.72 (0.05)	0.85 (0.03)	0.74 (0.05)
***Mentha aquatica***	0.92 (0.03)	0.83 (0.09)	0.96 (0.09)	0.71 (0.16)	0.76 (0.12)	0.83 (0.17)	0.76 (0.12)	0.83 (0.17)
***Mentha suaveolens***	0.80 (0.02)	0.69 (0.04)	0.68 (0.10)	0.65 (0.07)	0.80 (0.08)	0.78 (0.05)	0.78 (0.06)	0.77 (0.03)
***Rubus fruticosus***	0.67 (0.01)	0.61 (0.03)	0.64 (0.21)	0.62 (0.16)	0.64 (0.21)	0.58 (0.18)	0.64 (0.05)	0.60 (0.05)
***Rumex crispus***	0.82 (0.03)	0.82 (0.02)	0.81 (0.05)	0.82 (0.02)	0.76 (0.08)	0.75 (0.03)	0.76 (0.07)	0.76 (0.03)
***Scirpoides holoschoenus***	0.86 (0.03)	0.82 (0.02)	0.80 (0.09)	0.74 (0.05)	0.82 (0.10)	0.81 (0.06)	0.82 (0.08)	0.80 (0.05)
***Sorghum halepense***	0.72 (0.11)	0.61 (0.05)	0.62 (0.12)	0.72 (0.12)	0.80 (0.09)	0.57 (0.18)	0.80 (0.09)	0.58 (0.17)

Mean AUC values, positive predictive values, negative predictive values and overall accuracy were obtained by k-fold cross-validation (k = 4).

*M*. *aquatica* had the highest positive predictive value (0.96) and *S*. *halepense* the lowest (0.62) with the Maxent model (Table 3). *L*. *salicaria* had the highest positive predictive values (0.90) and *R*. *fruticosus* had the lowest (0.62) with the GLM model. *E*. *arvense* had the highest negative predictive value (0.86) and *E*. *juncea* the lowest (0.54) with the Maxent model. *M*. *aquatica* had the highest negative predictive value (0.83) and *E*. *juncea* the lowest (0.48) with the GLM model. For the Maxent model, the overall accuracy was then the highest for *E*. *arvense* and *L*. *salicaria* (0.85) and was low for *E*. *juncea* (0.6) and *R*. *fruticosus* (0.64). For the GLM model, the overall accuracy was the highest for *M*. *aquatica* (0.83) and the lowest for *E*. *juncea* (0.55) and *S*. *halepense* (0.58).

Based on Maxent and GLM models, the coefficients for each explanatory variable are presented in Tables 4 and 5. For each model, variables with high importance were considered those with coefficient values above the statistical median of the whole set of values attributed to the coefficients; the absolute value of the median was 9 for Maxent models and 2 for GLM.

Regarding the Maxent models, the importance was greater for **Doutlet** and **Mrvbf** than for the other variables for most of the 10 species. **Drain, Dnat,** and **Droad** were of secondary importance for Maxent models, and **Northness**, **Solar,** and **Slope** were of slight importance except for a few species. Geomorphological variables were then good explanatory variables for most species. **Mrvbf** was important for *A*. *acutifolius*, *E*. *arvense*, *L*. *salicaria*, *S*. *holoschoenus*, *M*. *aquatica*, *R*. *crispus*, and *S*. *halepense*. **Drain** was important for *L*. *salicaria*, *E*. *juncea*, *R*. *fruticosus*, *M*. *aquatica*, *S*. *halepense*, *and S*. *holoschoenus*. **Solar** was important for *A*. *acutifolius* and *R*. *fruticosus*, and **Northness** was important for *S*. *holoschoenus* and *M*. *aquatica*. **Slope** was important for *E*. *juncea* and *A*. *acutifolius*. Regarding distance to natural lands and distance to roads, **Dnat** was important for *R*. *fruticosus*, *E*. *arvense*, *M*. *aquatica*, *S*. *halepense*, and *R*. *crispus*. **Droad** was important for *S*. *halepense*, *M*. *suaveolens*, *R*. *fruticosus*, *E*. *arvense*, and *E*. *juncea*. **Dnat** and **Droad** had low importance for *S*. *holoschoenus*, *L*. *salicaria*, and *A*. *acutifolius*.

**Table 4:** Results for Maxent models for each species. Coefficients represent the relative importance of the explanatory variables (the sum of coefficients for each species is equal to 100). The more the coefficient is close to 100, the more the relative importance of the variable is high, compared to other variables.

Species	Doutlet	Drain	Mrvbf	Northness	Slope	Solar	Dnat	Droad
***Asparagus acutifolius***	18.1	0.9	43.9	0.4	9.2	17	5.8	4.6
***Elytrigia juncea***	42.4	13.7	4.8	2.0	9.2	7.2	8.9	11.8
***Equisetum arvense***	15.7	5.2	34.3	2.4	3.2	1.3	25.5	12.3
***Lythrum salicaria***	19.2	27.1	34.3	2.8	2.2	3.0	6.9	4.4
***Mentha aquatica***	35.8	11.2	16.4	9.8	1.2	1.7	21.4	2.5
***Mentha suaveolens***	58.1	5.8	4.6	1.7	2.6	4.8	4.9	17.5
***Rubus fruticosus***	17.5	13.2	2.4	2.5	6.0	16.7	29.3	12.4
***Rumex crispus***	46.3	8.9	16.4	1.7	2.4	5.3	11.5	7.5
***Scirpoides holoschoenus***	31.0	10.8	19.4	14.2	7.9	3.0	6.1	7.6
***Sorghum halepense***	33.1	10.8	9.9	4.0	3.8	1.1	18.6	18.6

**Table 5:** Results for GLM for each species. Regression coefficients are presented; their absolute value indicate the relative importance of the explanatory variables because explanatory variables have been rescaled between [0–1] before modelling. Only coefficients with p-value>0.05 were presented.

Species	Doutlet	Drain	Mrvbf	Northness	Slope	Solar	Dnat	Droad
***Asparagus acutifolius***	-	-	-2.3	-	3.3	1.3	-3.3	1.3
***Elytrigia juncea***	-	0.7	-	-	-5.3	-	0.7	-1.2
***Equisetum arvense***	-	-	2.7	-	-7.8	-	-4.2	-1.7
***Lythrum salicaria***	-2.4	3.3	5.2	-	-	-	-1.7	-1.6
***Mentha aquatica***	-	3.1	3.2	-	-	-	-3.1	-
***Mentha suaveolens***	-3.0	-	0.8	-	-	-	-	-2.8
***Rubus fruticosus***	0.5	0.5	-	-0.3	-2	-0.8	-1.4	-0.8
***Rumex crispus***	-2.4	1.3	1.6	-	-	-	2.3	-2.0
***Scirpoides holoschoenus***	-1.7	2.9	1.3	-1	-	-	-	-4.2
***Sorghum halepense***	1.3	-	-	-	-	-	-	-3.7

Regarding GLMs, **Droad**, **Dnat**, **Mrvbf**, and **Slope** were more important than the other variables for most of the studied plant species. **Doutlet** and **Drain** were of secondary importance for GLM models, and **Northness** and **Solar** were of low importance. Regarding correlations between geomorphological variables and species presence, **Mrvbf** was positively correlated with *L*. *salicaria*, *M*. *aquatica*, and *E*. *arvense* and was negatively correlated with *A*. *acutifolius*. **Slope** was negatively correlated with *E*. *arvense*, *E*. *juncea*, and *R*. *fruticosus* and positively correlated with *A*. *acutifolius*. **Doutlet** was negatively correlated with *M*. *suaveolens*, *R*. *crispus*, and *L*. *salicaria*. **Drain** was positively correlated with *L*. *salicaria*, *M*. *aquatica*, and *S*. *holoschoenus*. *S*. *halepense* occurrence was poorly explain by all of the geomorphological variables. Regarding distance to natural lands and roads, **Droad** was negatively correlated with *S*. *holoschoenus*, *S*. *halepense*, *M*. *suaveolens*, and *R*. *crispus*. **Dnat** was negatively correlated with *E*. *arvense*, *A*. *acutofolius*, and *M*. *aquatica* and was positively correlated with *R*. *crispus*. Geomorphological variables were then important to explain the distribution of all species with GLM, except for *S*. *halepense*.

**Fig 2:**
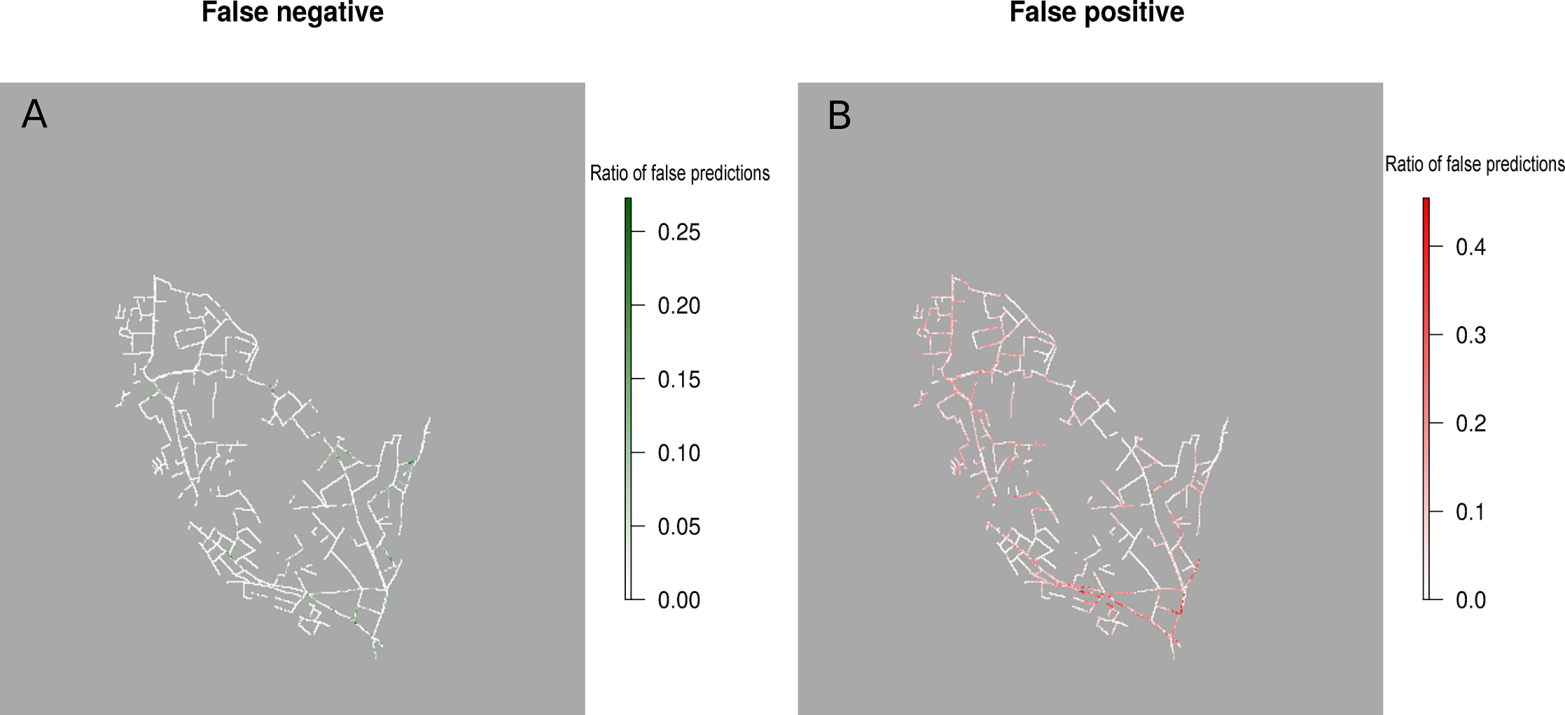
**Location of false negative (A) and false positive (B) predictions for Maxent model.** For each pixel, the value is the ratio between the number of false negative (2A), or false negative (2B) predictions for all species and the total number of species.

The residual maps revealed false positive predictions along major road axes in the southern catchment (Fig 2B). No spatialized tendency was evident, however, for false negative predictions (Fig 2A).

## Discussion

### Effect of geomorphological variables and distance to natural areas and roads

That geomorphological variables greatly affect plant spatial distribution has been previously demonstrated in Mediterranean streams [29] and in wetlands [26], but has not been previously assessed in ditches. This is reasonable for ditches because geomorphological features control the water regime and consequently greatly influence the fluxes of sediments, nutrients, pesticides and plant propagules that reach the ditches. Additionally, the results of the current study showed that the landscape geomorphological variables were at least as important as local geomorphological variables for determining the spatial distribution of plants. In agricultural landscapes, weed dynamics have often been linked to local features, but as reported by Alignier et al. [89] for in-field weeds, the factors controlling plant distribution can involve several interacting spatial scales. The dependence of plant ditch communities on the landscape scale has been previously shown for a land-use mosaic [33] but not for landforms. The results of the current study also highlighted the upstream-downstream gradient of the communities in the network, in that **Doutlet** was important for explaining the occurrence of all species. The weak ability of local geomorphological variables to predict species spatial distribution could also be explained by the expected greater error for local than for landscape variables, although the 6 geomorphological variables were derived from the DTM and DSM and calculated at the same resolution. Indeed, **Slope** calculation was based on the elevation difference between two points (at the beginning and end of the ditch) for which the uncertainty could be significant. Regarding **Solar**, due to the number of hypotheses we made (especially the fact that the pixel covered by vegetation was considered having no direct solar radiation), and due to the incertitude of measures of the geometrical properties of the ditch considered in the calculation, we can also expect it is less accurate than other variables calculated directly from the DTM and DSM.

In any case, the fact that landscape geomorphological variables, and especially **Drain**, **Doutlet** and **Mrvbf** were important predictors, shows indirectly the role of the hydrological connectivity at the catchment scale to explain the distribution of plant species in the ditches. As the modification of the geomorphology of the catchment is not possible, agro-ecological measures aiming at controlling the occurrence of plant species in Mediterranean agricultural landscapes should consider modifying this hydrological connectivity, for example by changing the density of the ditch network. Indeed, it has been confirmed in Levavasseur et al. [42] that the modification of the density of the network would have consequences on the water fluxes (especially the drained surface area and the peak discharge at the outlet) at the catchment scale in rain-fed Mediterranean agricultural catchments. In addition, the fact that the **Mrvbf** is also an important variable gives credit to measures affecting the sedimentological connectivity of the catchment, such as the optimization of the spatial distribution of vegetative filter strips involved in sediment trapping [90], to manage the occurrence of plants species.

Previous studies have demonstrated that land use can influence plant distribution in field boundaries by acting as seed bank sources or vectors [30,33,39]. Van Dijk et al. [34] reported that the occurrence of most species decreased with distance to nature reserves. Similar results were found in the present study as indicated by the negative correlation between **Dnat** and the occurrence of most species in GLM models. The strength of the association between the occurrence of species and the distance to seed sources or vectors was previously linked to the preferential dispersal strategy of the plant [33,34], but our data did not contain enough plant species to confirm this association. The effect of road proximity in our results was well-illustrated for *S*. *halepense*, which was previously shown to be dispersed with the movement of agricultural equipment [91]. These results illustrate the fact that measures based on the establishment of new seed bank sources such as vegetative filter strips (sown or not) along the ditches could be considered in order to enhance the occurrence of some species.

### Non-explained variability

The non-explained variability in spatial distribution varied greatly among the species in the current study. The selected explanatory variables were poor predictors of the spatial distribution for some species, especially for *R*. *fruticosus*, *S*. *halepense*, and *E*. *juncea*. The spatial variability of these species may be explained by factors that were not considered in our study. These factors could include biotic and abiotic variables and also the human activities involved in the maintaining of ditches.

Two main biotic factors that affect the spatial distribution of plants are dispersal processes and interspecific interactions such as competition and facilitation. Dispersal processes were indirectly taken into account in the current study by calculating the extent of spatial autocorrelation and by the choice of explanatory variables. However, competition and facilitation are rarely assessed in SDM studies [92]. These interactions are difficult to measure because variables that are considered to be biotic are sometimes micro-niche abiotic variables missing in the chosen pool of explanatory variables [93], and this may introduce collinearity effects between abiotic and biotic variables in the analysis [92]. Interactions would also be difficult to assess with the current data because they described the occurrence but not the abundance of the 10 selected species. Additionally, the influence of competition might be less important in drainage networks than in natural ecosystems because the networks are frequently disturbed [39,94]. However, these effects of competition and their importance can vary according to species. For example, Arnold et al. [95] showed that the perennial *S*. *halepense* had lower germination rates when the canopies of others plants were already developed, while the development of *R*. *fruticosus* was poorly impaired by the shading effects [96]. The consideration of these interactions represents a major challenge in the use of SDMs.

Abiotic variables from the environmental niche that could not be represented clearly by geomorphological variables were especially those linked with soil properties (e.g., pH, texture, structure, etc.). Firstly, the soil horizons in ditches have been formed over centuries when the geomorphology of the catchment was maybe slightly different. This could have implied different preferential places of deposition for sediments. The formation of the soils in the ditches was also influenced by the management practices led in the ditch network and in the plots of the upper catchment because agricultural practices affect the rates of transported materials [97]. Consequently, the current geomorphology partly controls where sediments are deposited today but soil formation in ditches was a long and multi-variable process, as was described in Needelman et al. [98], that is difficult to assess with static variables.

Another major challenge for SDM is the temporal variability of the explanatory variables. In the study area, the intermittency of rainfalls throughout the year and the inter-annual variability in the climate can create contrasted environmental conditions over time that could not be taken into account in our approach. This temporal variability can also affect plant interspecific relationships [99–100]. In some semi-arid landscapes, perennials create favourable or unfavourable conditions for the growth of annuals [101] depending on the environmental conditions, and especially according the water stress. The temporal variability in the explanatory variables is then a major challenge that could be partly solved taking into account the recent history of the explanatory variables, as was proposed in Alignier et al. [89] for management practices. However, for abiotic conditions, the problem of the temporal scales and types of indicators that we should use to take into account the recent history still needs to be solved.

The effects of ditch management practices have not been taken into account in this study. Yet, these management practices regularly disturb the ecosystem removing all the vegetation in place [14] and highly affect the local properties of the ditches [7]. It has been shown that both the type of practice and the time of the practice could affect the plant communities. In the Netherlands, a proper management of the ditch banks helped increase the richness of plant species [22]. It has also been shown that when there was a match between the period of the management practice and the seed maturity, the seed dispersal was optimal [38]. Consequently, for Mediterranean ditches, it would be interesting to know more about how these practices interact with plant species to understand better their spatial distribution.

The concentration of false positive predictions in the southern part of the study area and next to main roads remained unclear. These drainage networks are located near an urban area. In this suburban area, diffuse contaminants or non-agricultural maintenance practices may affect the spatial distribution of plants in ditches. Another explanation would be that these main roads would be less used by farm equipments, thus reducing the seed dispersal for species using this dispersal strategy.

These data on the sensitivity to explanatory variables could help guide the management of plants species in ditches in agricultural areas and in the suburbs of rural cities. For those species that are mostly explained by geomorphological variables, modifying the hydrological and sedimentological connectivity could be an effective way to manage their occurrence. For species mainly explained by distance to natural areas and distance to roads variables, supplementing seed bank sources on adjacent lands (by creating vegetative filter strips for example) would probably be effective. The factors that control the occurrence of species that were poorly predicted in this study need to be investigated. Future research should try to include the abundance of plants species, the whole community composition as well as management practices applied to the ditches.

### Performance of the Maxent model and GLM

Consistent with previous results [46, 67, 102], the Maxent model provided better spatial predictions for occurrence of plant species than the GLM model. The difference between Maxent and GLM performance was significant for the AUC but was slight for the other metrics. The stability of the predictions was correct because there was a moderate standard deviation (equal to or below 0.10) for all metrics after cross-validation, except for *S*. *halepense*, probably due to the small number of occurrence pixels after considering SAC or other explanatory variables that were not considered in the study, such as the sensitivity of some seeds to anthropogenic dispersion with agricultural equipment [103]. The ability of the Maxent model to consider interactions can also explain some of the differences in the identification of the main explanatory variables by the Maxent model vs. the GLM. Finally, the better performance of Maxent model than GLM may be explained by differences in data format. The GLM considers presence-absence data, while the Maxent model considers presence data compared against the entire region [71]. Although the sampling procedure of plant species was led at the whole catchment scale, the true absence of a plant species may be difficult to prove. Absence may reflect an unsuitable habitat or a suitable habitat that has not yet been colonized, as emphasised by Jarnevich et al. [104]. Other reasons for the absence of a species could be linked with the timing of the dispersal events [38] that can be variable according to the years, the variability of the conditions needed for the germination and establishment of the species, interspecific interactions and the specific management practices of the area (for example, the practice of dredging removes all the seed bank contained in the superficial soils [7]). Additional surveys on several years would be necessary to confirm the true absence of a species.

## Conclusions

This study illustrates the importance of considering geomorphological variables and the distance to natural lands and roads in order to predict the occurrence of some plant species living in agricultural drainage ditches in a rain-fed Mediterranean catchment. The importance of the landscape geomorphological variables outlines the role of the landscape environment surrounding a ditch in explaining the occurrence of these plant species. Consequently, agro-ecological management in Mediterranean ditch networks should investigate measures based on the modification of the hydrological and sedimentological connectivity in the catchment, such as the modification of the density of ditches and the optimization of the spatial organization of vegetative filter strips. Moreover, these vegetative filter strips could constitute new seed bank sources for ditch plants because the distance to seed bank sources and the distance to seed dispersal vectors (roads) were also important predictors in the study.
